# Factors influencing the health-related quality of life among persons with lower limb amputation wearing a prosthesis

**DOI:** 10.1192/j.eurpsy.2024.441

**Published:** 2024-08-27

**Authors:** C. Papathanasiou, A. Psoma

**Affiliations:** ^1^Department of Psychology, Panteion University of Social and Political Sciences, Athens; ^2^School of Social Sciences, Hellenic Open University, Patras, Greece

## Abstract

**Introduction:**

Limb amputation is often an unavoidable process in many diseases and accidents, leading to several limitations in social, professional, and recreational activities.

**Objectives:**

To explore the perceptions of persons with lower limb amputation (PLLA) wearing a prosthesis regarding the health-related quality of life (HRQoL), and to examine the relationships between HRQoL, body image disturbance, and self-esteem.

**Methods:**

The research sample consisted of 91 PLLA who were using a prosthesis. The data were collected through a questionnaire comprised of demographic information and the following scales: The Short Form Health Survey-12 (SF-12), the Amputee Body Image Scale (ABIS-R), and the Rosenberg scale (RSES), in order to assess HRQoL, body image disturbance, and self-esteem respectively. The SPSS statistical software (v.26) was used for the statistical analysis of the data.

**Results:**

The mean SF-12 score of the participants was 70.31 (SD=16.74). The HRQoL was affected by the following sociodemographic factors: age, educational level, profession, income, marital status, and parenthood. It was also influenced by disability-related factors, such as amputation cause and years of prosthesis use. In particular, young participants reported a better level of HRQoL than the older participants (p<0.001). Participants with a higher education level presented better HRQoL than those with lower education level (p<0.001). Unemployed participants and students presented better HRQoL scores compared to all other professional categories (p=0.001). However, participants with lower incomes <10,000 € reported a lower level of HRQoL (p=0.028). Singles had the highest HRQoL score, while widowers had the lowest (p=0.001). Childfree participants experienced the highest level of HRQoL (p=0.001). Participants whose amputation resulted from an accident reported a better HRQoL compared to those who had an amputation due to Type 2 diabetes (p<0.001). As the years of prosthesis use increase, HRQoL decreases (p=0.001). Regarding the associations between HRQoL, body image disturbance, and self-esteem statistically significant relationships were recorded. More specifically, there is a significant positive relationship between RSES and SF-12 (p<0.001); as participants’ self-esteem increases, so does their HRQoL. Conversely, a statistically significant negative correlation emerged between SF-12 and ABIS-R (p<0.001); as HRQoL increases, body image disturbance decreases.

**Conclusions:**

The aforementioned factors should be considered in the design and implementation of psychosocial interventions aimed at recovery. Qualitative studies are recommended to explore the lived experiences of PLLA in-depth.

**Disclosure of Interest:**

None Declared

